# Effects of age-based stereotype threat on time-based prospective memory

**DOI:** 10.3389/fpsyg.2024.1379160

**Published:** 2024-04-04

**Authors:** Alex Pak Lik Tsang, Stephen Cheong Yu Chan, Hui Jing Lu, Chi Chung Wong

**Affiliations:** ^1^Department of Applied Social Sciences, The Hong Kong Polytechnic University, Kowloon, Hong Kong SAR, China; ^2^Felizberta Lo Padilla Tong School of Social Sciences, Saint Francis University, Tseung Kwan O New Town, Hong Kong SAR, China

**Keywords:** age stereotype threat, older adults, prospective memory, time-based prospective memory, cognitive resource

## Abstract

The present study aimed to investigate the effect of a blatant activation of age-based stereotype threats (ABST) on time-based prospective memory (TBPM) in older adults. A sample of 74 adults from Hong Kong was randomly assigned to one of the two experimental conditions: the stereotyped condition (*n* = 36) or the neutral condition (*n* = 38). Participants were asked to read fictitious news reports related to dementia (stereotyped condition) or the importance of English oral skills (neutral condition). After, all participants performed a TBPM task using the Chinese lexical decision task as an ongoing task block. The results indicate a main effect of ABST on TBPM accuracy. Specifically, older adults under a blatant activation of ABST demonstrated lower TBPM accuracy (*p* < 0.05, *ηp^2^* = 0.08). Further analyses based on age groups demonstrated that TBPM accuracy was only impaired in older participants (aged 70–80 years) (*p* < 0.05, *ηp^2^* = 0.19). The study, for the first time, provides evidence that ABST can disrupt TBPM performance in older adults, especially when cues are blatantly activated.

## 1 Introduction

Prospective memory (PM) is an important form of memory that has been traditionally overlooked within the memory literature (Tsang et al., 2022). PM refers to the capacity to plan and execute future intentions under appropriate conditions. The literature generally distinguishes two types of PM: event-based (EBPM; i.e., an intention that is activated by the presence of an external cue, e.g., turning off the stove after cooking) and time-based (TBPM; i.e., an intention that is triggered after a certain amount of time has elapsed, e.g., attending a meeting after 2 h) (Einstein and McDaniel, 1990). Within the PM literature, TBPM often receives less attention than EBPM. However, TBPM is essential for older adults to sustain functional independence, such as taking medications on a regular schedule.

An intriguing phenomenon, often referred to as the age-PM paradox, has emerged in PM studies investigating the aging effects of PM. While older adults typically underperform young adults in PM tasks under experimental settings, they often outperform younger adults in more naturalistic PM tasks (Rendell and Thomson, 1999). Recently, the concept of age-based stereotype threats (ABST) has garnered significant attention within the domain of cognitive aging, with the potential to provide insights into explaining the age-PM paradox. Steele and Aronson (1995) originally described stereotype threat as a situation in which there is a risk to confirm a self-characteristic, negative stereotype associated with one’s group. In the context of ABST, the prevailing stereotypical view is that older adults are less cognitively competent (Fiske et al., 2002). This could have triggered ABST when older adults are being evaluated, particularly under experimental settings, and subsequently led to their underperformance. Although the effects of ABST have been reliably observed in the domains of episodic memory and working memory (for a meta-analysis, see Armstrong et al., 2017), it remains unclear whether ABST could differentially impact PM.

Given the significance of PM in supporting daily life activities and sustaining functional independence in older adults (Woods et al., 2012, 2015), to our knowledge, only one study has been conducted so far to investigate the effects of ABST on EBPM. Using a mixed factorial design, Zuber et al. (2019) compared the effects of ABST on PM cue focality between young and older adults. According to the Multi-Process Framework (McDaniel and Einstein, 2000), PM cue focality is a critical determinant of the mode of retrieval in EBPM. In focal EBPM, cues are aligned with the processing of an ongoing task (OT), and therefore detecting such cues would rely on a spontaneous retrieval process with minimal attentional effort. In contrast, retrieving a non-focal EBPM cue would require top-down strategic monitoring because it shares little processing features with the OT. Regarding ABST activations, they manipulated task instructions with one group emphasizing the mnemonic component of their experimental tasks (stereotyped condition), whereas the other group was told to evaluate their reading comprehension skills (neutral condition). They found that older adults have performed worse than younger adults only in the stereotyped condition under the more cognitively challenging non-focal EBPM task, suggesting that ABST could impact the age effect for cognitive processes associated with top–down attentional control. In contrast to EBPM, TBPM requires a higher degree of self-initiation due to the lack of an external cue (Craik, 1986), and therefore ABST should have a detrimental effect on TBPM performance.

However, the effects of ABST can be complicated by the different approaches through which it is activated, such as a subtle or blatant approach. Subtle activation of ABST involves manipulating the context of the study, while blatant cue manipulation entails direct reference to the stereotyped group regarding performance disadvantages in a particular domain (Armstrong et al., 2017). Subtle and blatant cue manipulations of ABST are thought to exert their effects through different mechanisms. Lamont et al. (2015), for example, suggested that using a subtler approach could elicit negative emotional responses due to its ambiguity. According to the Integrated Process Model of Stereotype Threat Effects (Schmader et al., 2008), the negative emotional responses associated with stereotype threats impose demands on the executive control component of working memory, resulting in fewer cognitive resources available to execute the task. Hence, the effects of ABST should be more pronounced when utilizing a subtle cue manipulation in tasks with a higher level of cognitive demands, which appears to be consistent with the findings in Zuber et al. (2019), where the ABST effects were only apparent in the non-focal EBPM task.

The effect of how ABST using a more blatant approach could differentially impact PM among older adults is still unknown. When blatant cues are explicitly defined, participants should be less prone to distractive thoughts and are more motivated to avoid task failures (Stone and McWhinnie, 2008). According to the Regulatory Focus Theory (Higgins, 1999), individuals with a promotion focus are typically more sensitive to the presence of gains, whereas those with a prevention focus are more concerned with losses. When individuals are motivated to avoid task failures under stereotype threats, they tend to temporarily invoke a prevention focus and are therefore more sensitive to losses (Seibt and Förster, 2004). This is often accompanied by a narrow attentional focus and increases in task monitoring (Beilock et al., 2006; Zhu and Meyers-Levy, 2007). However, a detrimental effect due to ineffective strategy use can be seen when task structure does not align well with a prevention focus, such as in memory tasks subjected to a temporal constraint (Hess et al., 2009). Considering that temporal constraint is inherent to TBPM intentions, it is possible that a prevention focus will disrupt its performance.

To fill this knowledge gap, this study aims to investigate the effects of a blatant activation of ABST on TBPM in older adults. We hypothesize that older adults under ABST will perform significantly poorer in TBPM than those in the neutral condition.

## 2 Methods

### 2.1 Participants

An *a priori* power analysis conducted using G*Power 3.1 indicated that a sample size of 68 is required for conducting a multivariate analysis of variance (MANOVA) to detect a medium effect size with two levels in a between-subject factor, with a power of 80% at *p* = 0.05. The total sample consisted of 76 participants, with a mean age of 67.66 (*SD* = 5.02). Consistent with the demographic profile of the Hong Kong older population (Hong Kong Census and Statistics Department, 2023), most participants were female (67.1%). The inclusion criteria were Chinese older adults aged 60–80 years old without any cognitive and affective disorders. Participants were recruited using convenience and snowball sampling, and those who took part were invited to refer their friends and relatives to participate in the study. All participants received an HKD 100 supermarket gift card as a financial incentive. This study was reviewed and approved by the Institutional Review Board in the corresponding author’s institution.

### 2.2 Materials and procedures

First, eligible participants provided written consent to participate in the study. Two screening measures were utilized to assess cognitive and affective impairments. The Hong Kong version of the Montreal Cognitive Assessment (MoCA-HK; Nasreddine et al., 2005, Yeung et al., 2014) was used to measure cognitive functions in different domains, including executive functions, naming, verbal memory, 5-min delayed recall, attention, abstraction, and orientation, resulting in a total score of 30. We adopted a cutoff of 21 for the MoCA-HK. Additionally, we employed the Patient Health Questionnaire-9 (PHQ-9; Kroenke et al., 2001; Cheng and Cheng, 2007) to measure depression severity over a 2-week recall period. It contains 9 items and is rated on a 4-point Likert scale (0 = not at all, 3 = nearly every day). The total score of PHQ-9 is 27, with higher scores indicating greater severity of depressive symptoms. For the PHQ-9, we adopted a cutoff of 9. Of note, one participant scored below the cutoff on the HK-MoCA and was excluded from this study.

Then, participants were instructed to perform a Chinese modification of the lexical decision task (LDT; Tsai et al., 2006). The experimental stimuli were displayed using the E-Prime software (Psychology Software Tools, Pittsburgh, PA) (Psychology Software Tools Inc, 2016)on a 15.6-inch display (screen resolution: 1920 × 1,080) with color-coded response keys. In the LDT, a central fixation (“+”) appeared on a black screen for 500 ms, followed by a two-character string (75 pt. font) for 3,000 ms. The two-character string could either be a real word or a non-word (i.e., a reversed sequence of characters without any associated meaning). Participants were instructed to press the “Y” button to indicate a word or the “N” for a non-word. Once a response was recorded, the stimulus remained onscreen, so that each trial had a fixed trial duration of 3,500 ms. A total of 30 LDT trials were administered, including 15 practice trials. After performing the LDT, participants were randomly assigned to one of the two conditions: stereotyped or neutral. They were given fictitious news articles related to either dementia (in the stereotyped condition) or the importance of English oral skills (in the neutral condition). To evaluate their understanding of our experimental manipulation, they were required to complete a reading comprehension exercise consisting of 10 questions related to the article’s content.

**Figure 1:**
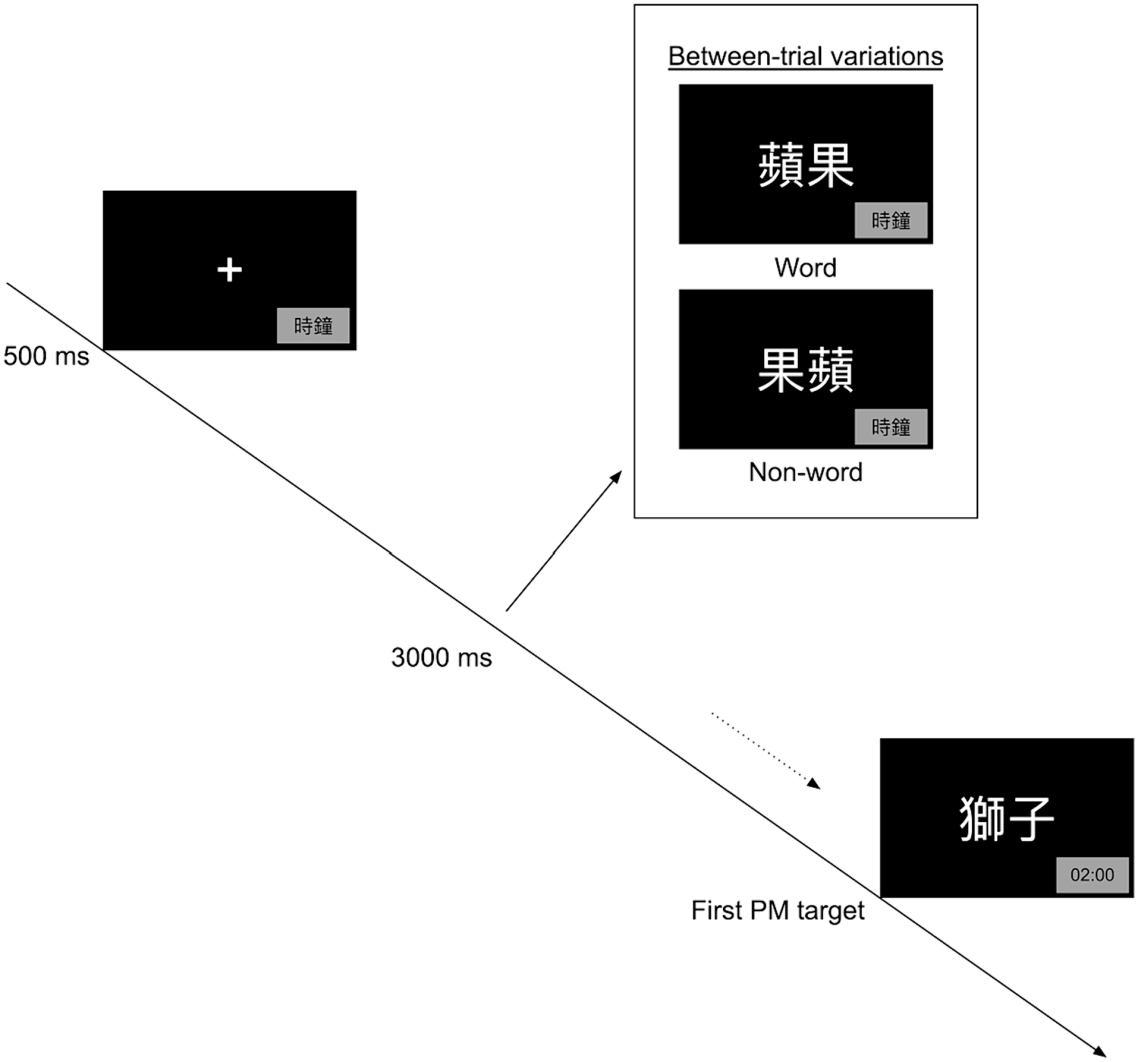
Procedures in the time-based prospective memory task. 時鐘 = clock, 蘋果 = apple, 果蘋 = the reversed sequence of “apple,” 獅子 = Lion.

**Figure 2:**
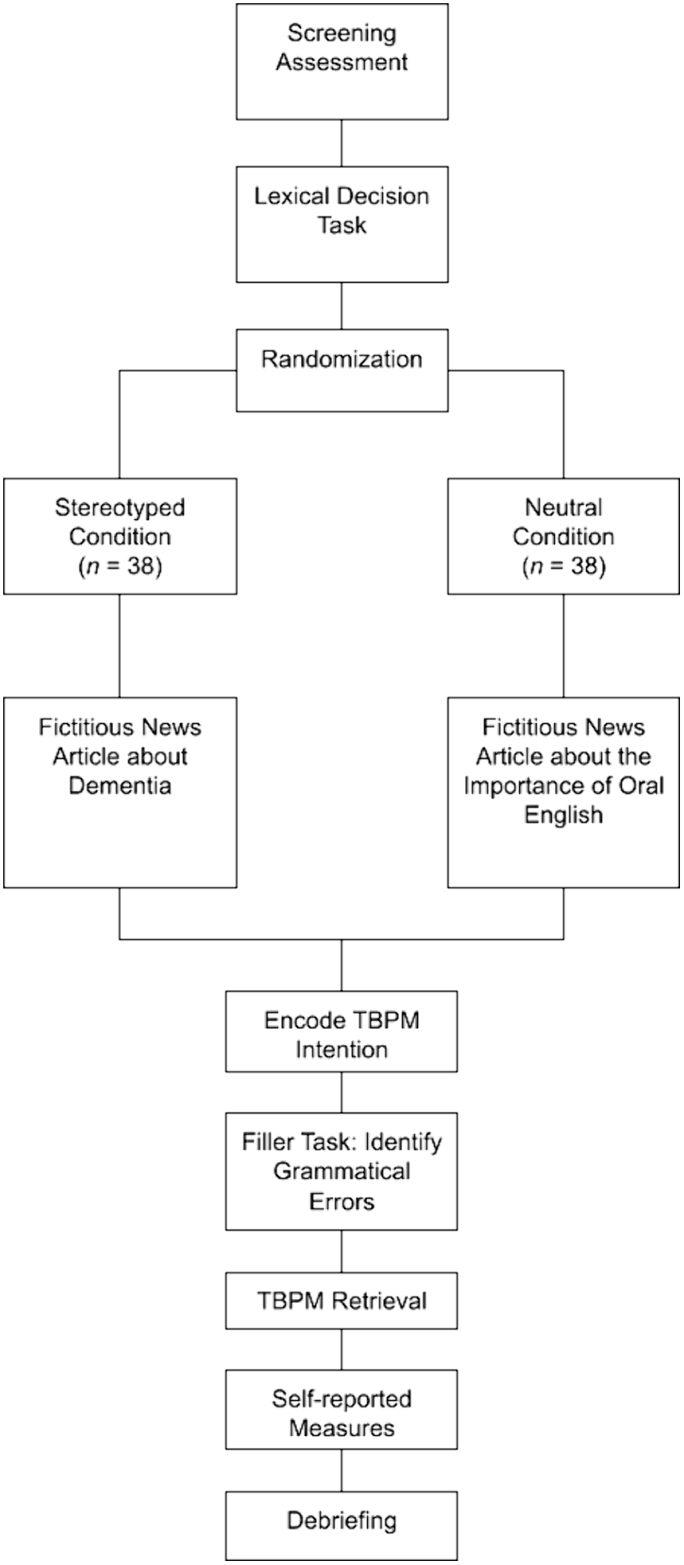
A flowchart of experimental procedures. TBPM, time-based prospective memory.

Next, participants encoded the TBPM intention. TBPM was assessed using the standard Einstein-McDaniel paradigm (Einstein and McDaniel, 1990). In this paradigm, TBPM commands were embedded in an OT, i.e., LDT. Specifically, participants were instructed to press the button “T” at a two-minute interval while performing the LDT. Additionally, they can press the button “C” to temporarily display a time counter, which appears for 3,000 ms at the bottom right corner of the screen. Once they had encoded the TBPM intention, participants completed a practice block consisting of two PM target intervals, lasting approximately 5 min. To induce PM forgetting, we implemented a filler task between the encoding and retrieval phases. During the filler task, participants were given an irrelevant article about the challenges in popularizing electric vehicles in Hong Kong, which contains 21 subtle grammatical errors. They were instructed to identify as many errors as possible within 5 min. After performing the filler task, participants retrieved the TBPM intention. The experimental block of TBPM consisted of 7 PM target intervals, lasting approximately 15 min (see Figure 1). Then, participants filled out self-reported socio-demographic questions. The experiment session ended with a debriefing session (see Figure 2). During the debriefing session, participants were provided with clarification regarding the study’s objectives, including the fact that the news they read during the study was fictitious and part of our experimental manipulation.

### 2.3 Data analysis

TBPM performance was quantified by the proportion of correct responses to PM targets and the number of accurate clock checks. A correct response in TBPM was defined by a time window of ±2,500 ms of each PM target interval (2 min). This time window has been widely adopted for two-minute PM target intervals in studies using the standard Einstein-McDaniel paradigm (Kliegel and Jäger, 2006; Jáger and Kliegel, 2008). However, this scoring revealed a flooring effect for both stereotyped (*M* = 0.36, *SD* = 0.31) and neutral conditions (*M* = 0.45, *SD* = 0.33), which may reduce variability and lead to an underestimation of the differences between the neutral and stereotyped conditions in TBPM performance. Considering the slowness of older participants, we adopted a more lenient scoring of ±15,000 ms in subsequent analyses (for a similar scoring approach, see Yang et al., 2013).

In addition, we also examined clock check behaviors. According to Joly-Burra et al. (2022), there are two general approaches to examining clock check behaviors in TBPM: the frequency of clock checks and the strategicness of clock checks. The overall frequency of clock checks refers to how often participants perform clock checking during the entire task block. Previous studies have associated more frequent clock checks with positive TBPM performance (Mioni et al., 2020). On the other hand, the strategicness of clock checks refers to the number of clock checks made 30 s before the PM target interval, as this timeframe of clock checks has been shown to be critical for executing an accurate TBPM response (Einstein et al., 1995). Therefore, in this study, we used both approaches to provide a more holistic understanding of TBPM performance.

Since PM performance could be influenced by the OT, we evaluated their LDT performance to determine if they comprehended the overall task instructions. Participants who scored less than 70% in accuracy were excluded from further analyses. Two participants were excluded on this basis, accounting for 2.6% of the total sample. Statistical analyses were based on the aim to explore differences in TBPM performance between the stereotyped and neutral conditions. Unless otherwise specified, statistical analyses did not exceed an alpha level of 5%.

## 3 Results

Socio-demographic variables were compared between the stereotyped and neutral conditions using t-tests or chi-square tests (see Table 1). No significant group differences were found. We also examined whether there were group differences regarding the performance on screening assessments (MoCA-HK and PHQ-9). The mean scores for MoCA-HK and PHQ-9 were 27.80 (*SD* = 1.92) and 1.91 (*SD* = 2.10), respectively. Again, no significant between-group differences were detected in the MoCA-HK, *t*(72) = 0.57, *p* = 0.57, or PHQ-9, *t*(66.77) = 1.31, *p* = 0.10. Next, we compared LDT performance between the stereotyped and neutral conditions. The proportion of correct responses and mean reaction time of the LDT was 0.94 (*SD* = 0.05) and 1410.03 (*SD* = 227.13), respectively. No significant group differences were observed in the proportion of correct responses, *t*(58.66) = 0.76, *p* = 0.45, or the mean reaction time, *t*(72) = 0.21, *p* = 0.83.

**Table 1:** Socio-demographic characteristics by conditions.

	Stereotyped condition (*n* = 36)	Neutral condition (*n* = 38)	*p*
Age, *M* ± *SD*	67.92 ± 5.33	67.29 ± 4.67	0.59^a^
Female, *n* (%)	23 (63.9%)	26 (68.4%)	0.68^b^
Education, *M* ± *SD*	3.22 ± 0.76	3.00 ± 0.66	0.18^a^
Employed, (%)	4 (11.1%)	3 (7.9%)	0.64^b^
Partnered or married, *n* (%)	16 (44.4%)	24 (63.2%)	0.11^b^

Education level was coded as 1 = preschool or below, 2 = primary, 3 = secondary, 4 = tertiary, 5 = postgraduate or above. *p*-values obtained by ^a^*t*-tests or ^b^chi-square tests.

As a manipulation check, we analyzed their mean scores in the reading comprehension exercise to determine whether participants comprehended the fictitious news articles. Participants who scored below 5 out of 10 were considered as unable to comprehend the articles, and thus not being successfully manipulated in ABST. Notably, one participant from the stereotyped condition scored below this cutoff and was excluded from subsequent analyses, accounting for 1% of the total sample. The mean scores for the stereotyped and neutral conditions were 9.40 (*SD* = 0.85) and 8.47 (*SD* = 1.35), respectively. An independent samples t-test further revealed a significant group difference, *t*(71) = −3.54, *p* < 0.01, suggesting that the reading comprehension exercise in the neutral condition could be more difficult than in the stereotyped condition. Considering that reading comprehension ability is required in the LDT, which may in turn affect TBPM performance, we controlled for their mean reading comprehension scores in subsequent analyses.

**Figure 3:**
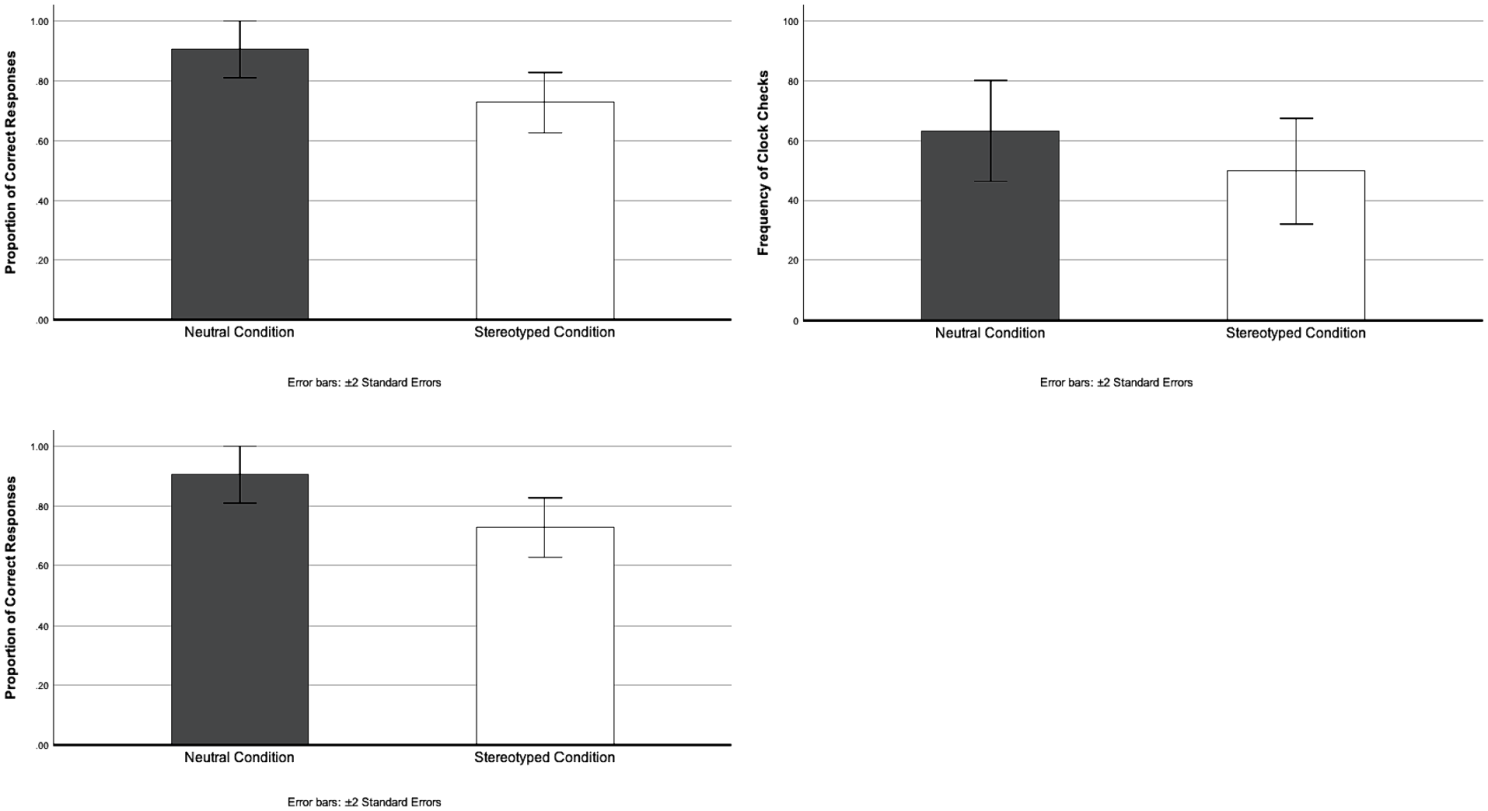
Performance in time-based prospective memory by conditions. TBPM, time-based prospective memory.

Performance in TBPM by conditions is presented in Figure 3. Before proceeding to the main analysis, correlational analyses were conducted to assess for multicollinearity among the dependent variables. Overall, no significant correlations were observed between reading comprehension and the proportion of correct responses (*r* = 0.12, *p* = 0.30), the frequency of clock checks (*r* = −0.16, *p* = 0.17), or the strategicness in clock checks (*r* = −0.04, *p* = 0.75). However, a weak positive correlation was identified between the proportion of correct responses and strategicness in clock checks in TBPM (*r* = 0.28, *p* < 0.05). Additionally, a strong positive correlation was detected between the frequency of clock checks and strategicness in clock checks (*r* = 0.92, *p* < 0.01), indicating that individuals who conducted more clock checks tended to exhibit greater strategic clock checking behavior in TBPM. Given the substantial correlations between the frequency and strategicness in clock checks, which could violate the multicollinearity assumptions in MANOVA, multiple univariate analyses of covariance (ANCOVA) were conducted to investigate the effects of ABST on each TBPM performance indicator while controlling for reading comprehension scores.

The ANCOVA results indicated a significant main effect of ABST on the proportion of correct responses in TBPM, *F*(1, 70) = 6.157, *p* < 0.05, *ηp^2^* = 0.08, whereas the influence of reading comprehension on response accuracy was marginally non-significant, *F*(1, 70) = 3.82, *p* = 0.06, *ηp^2^* = 0.05. These results suggested that participants in the stereotyped condition (*M* = 0.76, *SD* = 0.35) performed less accurately than those in the neutral condition (*M* = 0.88, *SD* = 0.22).

Regarding the frequency of clock checks, participants in the stereotyped condition (*M* = 65.24, *SD* = 55.76) conducted fewer clock checks compared to those in the neutral condition (*M* = 47.57, *SD* = 43.05). However, the results did not demonstrate a main effect of ABST, *F*(1, 70) = 1.12, *p* = 0.29, *ηp^2^* = 0.02. A similar pattern was observed in the strategicness of clock checks, with participants in the stereotyped condition (*M* = 19.43, *SD* = 14.43) displaying less strategic clock-checking behaviors than those in the neutral condition (*M* = 22.68, *SD* = 13.34). Nevertheless, the main effect of ABST was nonsignificant, *F*(1, 70) = 0.89, *p* = 0.35, *ηp^2^* = 0.01 (Table 2).

**Table 2:** Performance in time-based prospective memory stratified by age groups.

	Younger participants (*n* = 50)			Older Participants (*n* = 23)		
	Stereotyped condition (*n* = 22)	Neutral condition (*n* = 28)		Stereotyped condition (*n* = 13)	Neutral condition (*n* = 10)	
	*M* ± *SD*	*M* ± *SD*	*p (ηp^2^)*	*M* ± *SD*	*M* ± *SD*	*p (ηp^2^)*
Proportion of correct responses	0.81 ± 0.32	0.87 ± 0.24	0.39 (0.01)	0.66 ± 0.40	0.91 ± 0.15	**<0.05 (0.19)**
Frequency of clock checks	52.36 ± 49.67	73.68 ± 62.29	0.83 (<0.01)	39.46 ± 28.64	41.60 ± 17.34	0.70 (0.01)
Strategicness in clock checks	21.23 ± 16.15	24.54 ± 14.67	0.90 (<0.01)	16.38 ± 10.83	17.50 ± 6.79	0.64 (0.01)

One younger participant in the stereotyped condition was excluded. Bold value means the statistically significant (*p* < 0.05).

Considering previous studies have shown that the effects of ABST on EBPM vary depending on age (Zuber et al., 2019), we conducted additional stratified analyses to examine whether ABST had differential effects on TBPM performance among younger participants (aged 60–69 years) and older participants (aged 70–80 years). The stratified analyses revealed that the effect of ABST on the proportion of correct responses was only evident in older participants, *F*(1, 20) = 4.71, *p* < 0.05, *ηp*^2^ = 0.19, in that the stereotyped condition (*M* = 0.66, *SD* = 0.40) performed worse in TBPM compared to the neutral condition (*M* = 0.91, *SD* = 0.15). However, we did not observe any main effects of ABST on the frequency and strategicness of clock checks in both younger and older participants.

## 4 Discussion

The present study aimed to investigate the impact of ABST on TBPM in older adults, particularly when ABST is blatantly activated. The results confirm our hypothesis, indicating a main effect of ABST on TBPM accuracy. In other words, older adults subjected to a blatant activation of ABST exhibited lower TBPM accuracy. Although older adults in the stereotyped condition exhibited a tendency for reduced clock checks and less strategic clock check behavior, these outcomes did not reach statistical significance. Furthermore, our additional stratified analyses revealed that the influence of ABST on TBPM accuracy was only evident in older participants (aged 70–80 years). This finding aligns with the findings of Zuber et al. (2019), who found that older adults aged 71 years and above performed less accurately than younger adults in non-focal EBPM under a subtle ABST manipulation. Together, our results, in conjunction with those of Zuber et al. (2019), indicate a detrimental effect of ABST on PM performance in older adults, providing an alternative explanation for the age-PM paradox. This suggests that the lower PM performance observed in older adults during experimental PM tasks may be partly influenced by the effects of ABST.

Furthermore, our results confirm that, in addition to EBPM, TBPM is susceptible to the effects of ABST. Traditionally, TBPM has been understudied in the scant PM literature. However, there are significant implications regarding the effects of ABST on TBPM. For example, TBPM is integral to many specific tasks that are essential for older adults to maintain their functional independence, such as adhering to medication schedules (Woods et al., 2009). Furthermore, TBPM performance in older adults is positively associated with quality of life, with implications for successful aging (Woods et al., 2015). Given its significance on functional independence, it is common for older adults with subjective memory complaints to express concerns about TBPM. Therefore, when practitioners conduct clinical assessments of memory functioning, it is important to be cautious about whether ABST may play a role in obtaining an accurate account of actual memory capabilities.

Our results also partially support the notion that stereotype threats disrupt strategic memory processes. According to the Integrated Process Model of Stereotype Effects, negative emotional responses associated with ABST impose additional demands on working memory, leading to reduced availability of cognitive resources for task performance (Schmader et al., 2008). For example, Zuber et al. (2019) utilized a subtle manipulation of ABST and found that older adults performed worse than younger adults only in a more cognitively demanding non-focal EBPM task, which requires greater attentional resources for strategic monitoring compared to focal EBPM tasks that rely on spontaneous retrieval (McDaniel and Einstein, 2000). Due to the absence of external cues, TBPM should have the highest degree of self-initiation among different types of PM (Craik, 1986). Our age-stratified analyses further support this notion. Despite our modest sample size in the older subgroup aged between 70 to 80 years old (*n* = 23), we were still able to detect a large effect of ABST. This finding emphasizes that older participants, who may experience a decline in cognitive resources associated with aging (Kirasic et al., 1996), may encounter difficulties in strategic memory processes, particularly under the influence of ABST.

However, it is worth mentioning that the effects of ABST on clock-checking behaviors did not reach statistical significance. A potential explanation could be attributed to our blatant manipulation of ABST. When confronted with a blatant manipulation of ABST, older adults may activate a preventive focus and become vigilant in order to avoid confirming age-related declines (Barber, 2017). According to the Regulatory Focus Theory, individuals with a prevention focus are motivated to avoid losses, which often leads to an increased level of task monitoring (Higgins, 1999; Zhu and Meyers-Levy, 2007). Therefore, it is possible that the heightened level of task monitoring resulting from the temporal activation of a preventive focus may have increased clock-checking behaviors. As a result, we may have failed to detect any significant group differences, although there was a trend toward reduced clock checks and less strategic clock-checking behavior under the effects of ABST. On the other hand, the impaired TBPM accuracy under the influence of ABST could be attributed to ineffective strategy utilization. It is plausible that as ABST imposes additional demands on working memory, a significant amount of cognitive resources are allocated toward engaging in clock monitoring behaviors due to the temporal activation of a preventive focus. Consequently, there may be insufficient cognitive resources available for the successful retrieval of the TBPM intention. Given that the effects of ABST can be associated with increased physiological arousal, especially among older adults who perceive aging as a fixed and inevitable process (Weiss, 2016), further investigation could involve incorporating measures of physiological reactivity, such as systolic blood pressure, to confirm the effects of ABST on clock checking behaviors in TBPM.

There are several limitations in this study. First, the present study did not include younger participants, and therefore, it is difficult to delineate whether the observed ABST effect on TBPM is exclusive to older adults. In a younger population, the experimental manipulation in the stereotyped condition (reading fictitious news about dementia) might implicitly prime the notion that the subsequent TBPM task could be difficult. This could lead to inaccurate judgment of task difficulty in younger individuals, potentially resulting in ineffective cognitive strategies and impaired task performance (Vangsness and Young, 2019). Nonetheless, previous research has highlighted the importance of age group identification and the level of identification with one’s age group as moderating factors in the effects of ABST on cognitive performance (O'Brien and Hummert, 2006). Additionally, Zuber et al. (2019) observed that the effects of ABST on EBPM were only evident in older, but not younger adults. To further confirm the age-specific effects of ABST on TBPM, it is important for future studies to adopt a factorial design to explore the interaction effects between stereotype conditions and age groups on TBPM.

Second, the effects of positive age stereotypes on TBPM in older adults were not investigated in our study. Common positive attributes associated with aging include wisdom and knowledge (Baltes and Staudinger, 1993). Indeed, a longitudinal study has shown that older adults with positive self-perceptions of aging engage in more health behaviors over a 20-year period (Levy and Myers, 2005). In a seminal study conducted by Levy (1996), it was found that exposure to positive age stereotypes among older adults can lead to enhanced episodic memory performance and memory self-efficacy. In addition, the experience of ABST is a culturally-bound phenomenon (Luo et al., 2013). Tan and Barber (2018), for example, found that Confucian beliefs in filial piety can reverse the impacts of ABST on episodic memory. Considering the crucial role of PM capacity in older adults’ functional status, it is important to investigate the potential utility of positive age stereotypes as an intervention to counter prevailing negative stereotypes and improve PM performance. To our knowledge, no study has explored the effects of positive age stereotypes on PM. Therefore, we encourage future studies to explore this area and assess the influence of positive age stereotypes on PM.

Third, we did not explore the complex effects of intersectional stereotypes on TBPM. Intersectional stereotypes refer to the overlapping effects of multiple social identities that may interact in unique combinations (Crenshaw, 1989). Recently, there has been a surge of interest in exploring the intersectionality of social stereotypes, such as age-by-race police discrimination (Hester et al., 2020). In the domain of cognitive processes, there is a widely held belief that women outperform men in multitasking, partly due to a perceived developmental advantage in extensive multitasking practices such as managing children, family, and career (Szameitat et al., 2015). Moreover, the capacity for multitasking and PM are closely associated (Burgess et al., 2000), given that the dual-task nature of PM requires multitasking. Considering that positive stereotypes can lead to a performance boost on stereotype-relevant tasks (Oswald, 2008), it is possible that such a positive stereotype can result in an improvement in PM performance. Therefore, an age-by-gender intersectional stereotype may be at play, where the positive stereotype regarding gender on multitasking may mitigate the negative influences of ABST on TBPM, particularly for female participants. Given the complex nature of stereotype threats, future studies should explore whether the intersectionality of multiple social stereotypes interacts with ABST in influencing the performance of TBPM.

As PM is a multi-phased process, successful execution of PM tasks involves an encoding phase followed by the retention phase, and eventually the retrieval and execution phases (Ellis, 1996). Given that Zuber et al. (2019) and the present study activated ABST preceding the encoding phase, it remains unclear how ABST may differentially impact each PM phase. Using subliminal ABST cues, Krendl et al. (2015) found that the impacts on episodic memory were more pronounced when the cues were presented preceding the retrieval phase than the encoding phase. Therefore, future studies may directly address this aspect to provide further insights into the underlying mechanisms of ABST on PM.

Despite these limitations, this study confirms the effects of ABST on TBPM in an older population. According to the Integrated Process Model of Stereotype Effects, the effects of ABST may disrupt strategic memory processes. This is evident in our age-stratified analyses, where TBPM accuracy was specifically impaired in older participants (aged 70–80 years), indicating that older individuals may be more susceptible to ABST due to a decline in cognitive resources necessary for strategic memory processes. While the literature on ABST has predominantly focused on retrospective memory, the present study offers further support for the presence of ABST in PM.

## Data availability statement

The raw data supporting the conclusions of this article will be made available by the authors, without undue reservation.

## Ethics statement

The studies involving humans were approved by Ethical approval was obtained from the research ethics committee of Saint Francis University (formally the Caritas Institute of Higher Education) in accordance with the guidelines and procedures for ethical review regarding human research (REC Ref. no.: HRE230221). The studies were conducted in accordance with the local legislation and institutional requirements. The participants provided their written informed consent to participate in this study.

## Author contributions

AT: Formal analysis, Methodology, Writing – original draft, Writing – review & editing. SC: Conceptualization, Formal analysis, Funding acquisition, Investigation, Methodology, Supervision, Writing – original draft, Writing – review & editing. HL: Supervision, Writing – original draft, Writing – review & editing. CW: Project administration, Writing – original draft, Writing – review & editing.
